# Early Postoperative Remodeling of the Exhaled Volatilome Following Curative Surgery for Colorectal Cancer

**DOI:** 10.3390/jcm15186983

**Published:** 2026-09-09

**Authors:** Elizaveta Tyukanova, Zaki Fashafsha, Aleksandr Suvorov, Anastasia Fatyanova, Valeriy Ponomarev, Artemiy Silantyev, Denis Khlusov, Akmalbek Otabekov, Yaroslav Krasnov, Maksim Volgin, Bogdan Tkachenko, Albina Zubayraeva, Sergey Efetov, Philipp Kopylov, Marina Sekacheva

**Affiliations:** 1 Institute for Personalized Oncology, Biomedical Science & Technology Park, I.M. Sechenov First Moscow State Medical University (Sechenov University), Trubetskaya Str., Bldg. 8/2, 119991 Moscow, Russia; sekacheva_m_i@staff.sechenov.ru; 2 Oncology Department of Antitumor Drug Therapy, Sechenov University Clinical Hospital No.4, I.M. Sechenov First Moscow State Medical University (Sechenov University), Str. Dovatora, 15, Bldg. 2, 119048 Moscow, Russia; tkachenkobogdan96@gmail.com; 3 P. Hertsen Moscow Oncology Research Institute—Branch of the Federal State Budgetary Institution “National Medical Research Radiological Centre” of the Ministry of Health of the Russian Federation, 2nd Botkinsky Proezd, 3, 125284 Moscow, Russia; fashafshazaki@gmail.com; 4 Institute of Personalized Cardiology of Biomedical Science and Technology Park, I.M. Sechenov First Moscow State Medical University (Sechenov University), Trubetskaya Str., bldg. 8/2, 119991 Moscow, Russia; suvorov_a_ya_1@staff.sechenov.ru (A.S.); silantev_a_s@staff.sechenov.ru (A.S.); kopylov_f_yu@staff.sechenov.ru (P.K.); 5 The Department of Oncology, Radiotherapy and Reconstructive Surgery, I.M. Sechenov First Moscow State Medical University (Sechenov University), Trubetskaya Str., Bldg. 8/2, 119991 Moscow, Russia; fatyanova@mail.ru; 6 Oncology Department, N.V. Sklifosovsky Institute of Clinical Medicine, I.M. Sechenov First Moscow State Medical University (Sechenov University), Trubetskaya Str., Bldg. 8/2, 119991 Moscow, Russia; ponomarev_v_e@staff.sechenov.ru; 7 Department of Faculty Surgery No. 2, I.M. Sechenov First Moscow State Medical University (Sechenov University), Trubetskaya Str., Bldg. 8/2, 119991 Moscow, Russia; denis.khlusov@yandex.ru (D.K.); yarikkrasnov@gmail.com (Y.K.); zubayraeva_a_a@staff.sechenov.ru (A.Z.); 8 Surgical Department No. 2, University Clinical Hospital No. 4, Clinical Center, I.M. Sechenov First Moscow State Medical University (Sechenov University), Str. Dovatora, 15, Bldg. 2, 119048 Moscow, Russia; otabekov_a_a@staff.sechenov.ru (A.O.); maxvolgiy_m@mail.ru (M.V.); efetov_s_k@staff.sechenov.ru (S.E.)

**Keywords:** colorectal cancer, volatile organic compounds, breath analysis, proton transfer reaction time-of-flight mass spectrometry, PTR-TOF-MS, volatilome, curative surgery, postoperative volatilome remodeling, machine learning, VOC features

## Abstract

**Background:** Exhaled volatile organic compounds (VOCs) may provide a non-invasive readout of biological changes in colorectal cancer (CRC). We characterized early postoperative changes in the exhaled volatilome after curative CRC surgery and explored a multivariable VOC signature associated with clinical state. **Methods:** In this prospective paired pilot study, breath samples were obtained from 38 patients before surgery (Before) and on postoperative day 10 (After) and from 44 healthy controls (Control). Proton transfer reaction time-of-flight mass spectrometry (PTR-TOF-MS) identified 61 informative volatile organic compound (VOC) features. Elastic Net-based stability selection with 1000 patient-grouped resampling iterations yielded a 16-feature panel. Pairwise univariate comparisons were performed with Benjamini–Hochberg false discovery rate (FDR) correction. **Results:** Five selected VOC features differed significantly between paired preoperative and postoperative samples. Two features (mass-to-charge ratio [*m*/*z*] 77.0576 and 103.074) no longer differed significantly from healthy controls postoperatively, whereas three (*m*/*z* 63.0157, 95.0534, and 101.05) remained significantly different. The highest discriminative performance was observed for After vs. Control (median ROC AUC = 0.811 [95% PR, 0.691–0.912]), followed by Before vs. Control (0.762 [0.629–0.869]) and Before vs. After (0.716 [0.567–0.823]). **Conclusions:** Measurable changes in the exhaled volatilome were observed on postoperative day 10 following curative colorectal cancer surgery, with heterogeneous patterns across individual VOC features and persistent differences from healthy controls. These findings should be interpreted as early postoperative volatilome remodeling rather than as evidence of tumor-specific metabolic normalization because the observed changes may reflect the combined influence of surgical trauma, postoperative recovery, and other biological and perioperative processes. The identified 16-feature multivariable VOC signature should therefore be regarded as exploratory and requires independent external validation. Further studies incorporating larger multicenter cohorts, serial sampling beyond the early postoperative period, appropriate non-oncological surgical control groups, and orthogonal chemical identification are required to determine the potential clinical relevance of longitudinal VOC patterns.

## 1. Introduction

Colorectal cancer (CRC) remains one of the most common malignancies and a leading cause of cancer-related mortality worldwide. According to recent global estimates, approximately two million new cases and more than 900,000 deaths occur annually, highlighting its substantial healthcare burden [1,2]. Despite advances in screening, surgical techniques, systemic therapy, and perioperative management, CRC continues to impose considerable clinical and socioeconomic challenges. Furthermore, its global incidence is projected to increase owing to population ageing, the growing prevalence of lifestyle-related risk factors, and the rising incidence of early-onset disease, underscoring the need for more effective strategies for disease monitoring and postoperative surveillance.

Regardless of substantial advances in colorectal cancer screening and diagnostic technologies, important challenges remain in the early detection and longitudinal monitoring of the disease. Colonoscopy remains the reference standard for CRC diagnosis because it enables direct visualization of the intestinal mucosa and histopathological confirmation of suspicious lesions. However, its invasive nature, the need for bowel preparation, procedure-related discomfort, and limited adherence to screening programs restrict its effectiveness as a population-wide surveillance tool [3].

Cross-sectional imaging modalities, including computed tomography (CT) and magnetic resonance imaging (MRI), are indispensable for disease staging and treatment planning but primarily characterize anatomical changes rather than the underlying biological activity of the tumor. Likewise, currently available blood- and stool-based biomarkers, including carcinoembryonic antigen (CEA), carbohydrate antigen 19-9 (CA19-9), fecal immunochemical testing (FIT), and stool DNA assays, provide valuable complementary information but demonstrate limited sensitivity for early disease and incompletely reflect dynamic tumor-associated metabolic alterations [4,5,6].

These limitations have stimulated growing interest in functional biomarkers capable of capturing real-time biological changes during tumor development and treatment. Among emerging breathomics approaches, analysis of exhaled breath has attracted considerable attention because VOCs can reflect endogenous metabolic processes and may provide a non-invasive readout of tumor-associated metabolic reprogramming, offering new opportunities for disease detection and longitudinal monitoring of colorectal cancer [7].

Metabolic reprogramming is a hallmark of cancer and plays a central role in tumor initiation, progression, and adaptation to the tumor microenvironment [8]. Compared with normal tissues, malignant cells undergo profound alterations in glucose, lipid, and amino acid metabolism, accompanied by oxidative stress and mitochondrial dysfunction, which collectively generate a distinct metabolic phenotype associated with tumor aggressiveness and disease progression [9,10,11,12,13]. These metabolic processes give rise to a broad spectrum of VOCs, including aldehydes, ketones, hydrocarbons, and other low-molecular-weight metabolites produced through altered intermediary metabolism and lipid peroxidation [12,13,14,15,16,17,18,19,20]. Owing to their physicochemical properties, VOCs readily diffuse into the bloodstream and are subsequently excreted via the lungs, making exhaled breath an accessible source of metabolic information [17,19,20]. Because the exhaled volatilome may reflect contributions from malignant cells as well as stromal, immune, host, and microbial processes, it represents an integrated biological phenotype rather than the activity of a single biological pathway [21,22].

Unlike tissue biopsy or conventional imaging, breath analysis is entirely non-invasive, readily repeatable, and capable of capturing dynamic metabolic changes, making it particularly attractive for longitudinal disease assessment. Consequently, breathomics has emerged as a promising approach for identifying metabolic signatures relevant to colorectal cancer detection and longitudinal disease assessment [23,24,25,26,27,28,29,30,31,32,33].

Previous studies have demonstrated changes in exhaled VOC profiles following curative colorectal cancer surgery using different analytical approaches, including thermal-desorption GC-MS and electronic-nose technology [34,35]. These studies included both longer-term postoperative observations and prospective paired assessments during the early postoperative period. Thus, postoperative VOC changes themselves are not unique to the present study. Rather, the present investigation extends these observations by applying real-time PTR-TOF-MS to paired preoperative and postoperative day-10 breath samples and by characterizing early postoperative volatilome remodeling at the level of individual mass-to-charge features and an exploratory multivariable VOC signature. Among the analytical platforms available for breathomics, PTR-TOF-MS enables rapid, highly sensitive, real-time detection of a broad spectrum of volatile organic compounds with minimal sample preparation [36,37,38,39,40].

From a translational perspective, longitudinal breath profiling may provide a non-invasive and repeatable approach for assessing postoperative VOC dynamics, although its potential role in clinical surveillance remains to be established.

Therefore, the aim of this study was to characterize early postoperative changes in the exhaled VOC profile of patients with colorectal cancer following curative surgery using PTR-TOF-MS and to identify multivariable VOC signatures associated with these changes. To address this objective, we used a paired breath-sampling design integrating high-resolution PTR-TOF-MS profiling with multivariable statistical and machine-learning approaches and evaluated the discriminatory performance of the resulting VOC signature.

## 2. Materials and Methods

### 2.1. Study Design and Participants

A prospective, single-center, non-randomized, observational pilot cohort study with a paired longitudinal component was conducted at the University Clinical Hospitals of I.M. Sechenov First Moscow State Medical University (Sechenov University, Moscow, Russia). For patients with colorectal cancer, a paired breath-sampling design was used, with samples collected from the same individuals before curative surgery (Before) and on postoperative day 10 (After). Postoperative day 10 was selected as a standardized early postoperative sampling time point to enable paired assessment within a consistent clinical timeframe; it was not intended to represent resolution of the physiological effects of surgery. Patients with colorectal cancer were prospectively enrolled between March 2023 and July 2024. As this was an exploratory pilot study, no formal a priori sample-size calculation was performed; the analytical cohort was determined by prospective recruitment and availability of complete paired preoperative and postoperative breath samples.

The study enrolled adult patients with histologically confirmed locally advanced colorectal adenocarcinoma (clinical stage II–III), and an independent cohort of healthy volunteers. The diagnosis of CRC was established based on histopathological examination of the primary tumor in combination with endoscopic evaluation and radiological imaging, including contrast-enhanced computed tomography (CT) and magnetic resonance imaging (MRI). Serum tumor markers, including carcinoembryonic antigen (CEA) and carbohydrate antigen 19-9 (CA19-9), were assessed as part of the routine diagnostic work-up. Patients with CRC underwent radical surgery as the first stage of treatment in accordance with clinical guidelines.

**Figure 1:**
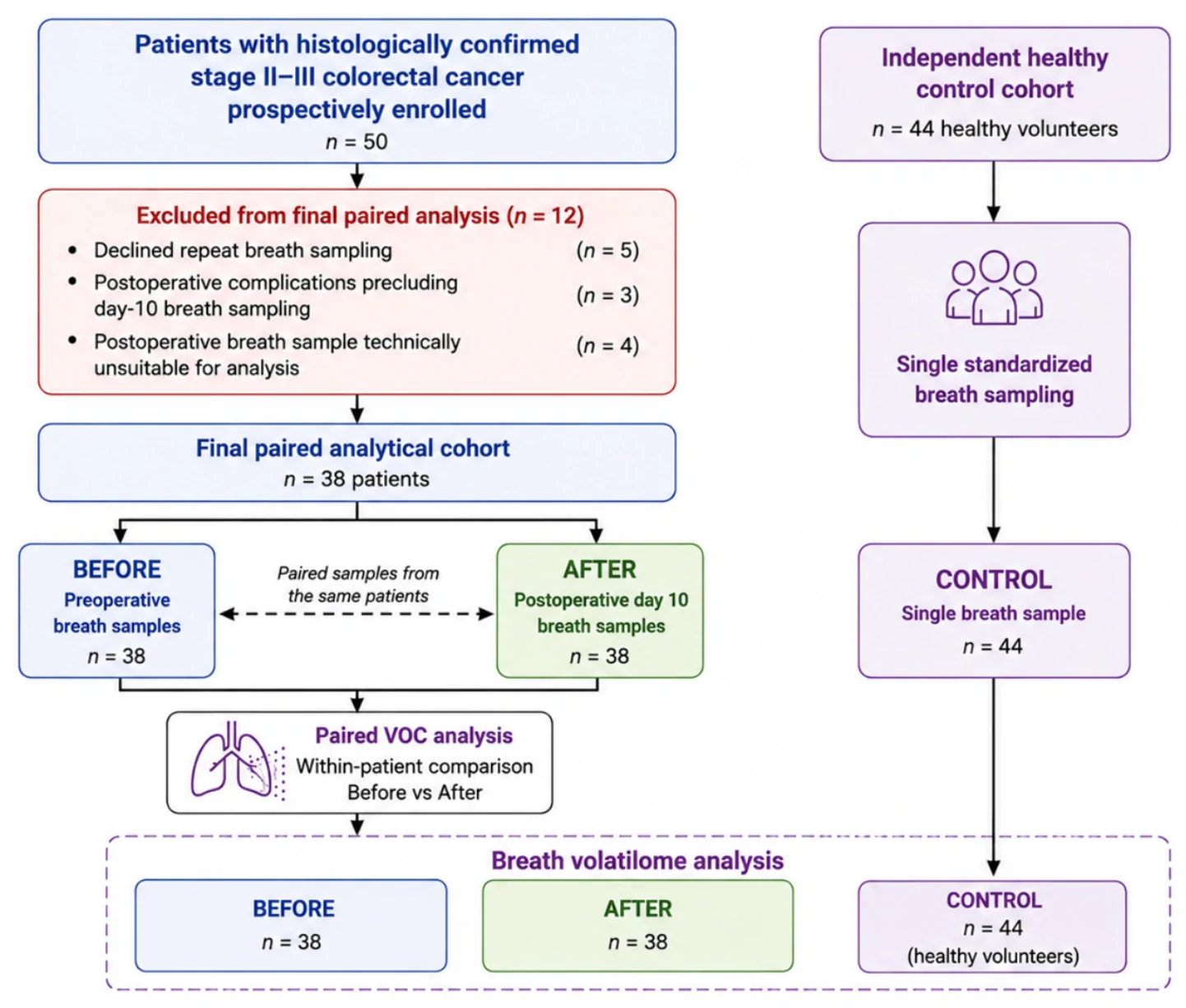
Study design and patient flow. Fifty patients with histologically confirmed stage II–III colorectal cancer were prospectively enrolled. Twelve patients were excluded from the final paired analysis because a valid postoperative day-10 breath sample was unavailable, resulting in a final paired analytical cohort of 38 patients with complete preoperative (Before) and postoperative day-10 (After) breath samples. The Control group comprised an independent cohort of 44 healthy volunteers who underwent a single standardized breath-sampling procedure.

Initially, 50 patients with histologically confirmed stage II–III CRC were prospectively enrolled. Of these, 12 patients were excluded from the final paired analysis because a valid postoperative day-10 breath sample was unavailable. Specifically, five patients declined repeat breath sampling, three developed postoperative complications that precluded breath sampling on postoperative day 10, and in four patients the postoperative breath sample was collected but was technically unsuitable for analysis. To preserve the paired analytical design, only patients with both valid preoperative and postoperative day-10 breath samples were included in the final analysis. Consequently, the final paired analytical cohort comprised 38 patients with CRC, contributing 38 Before and 38 After breath samples. Together with the independent healthy control group, these samples formed the three analytical groups used for breath volatilome analysis: Before, After, and Control. The overall study design and participant flow are presented in Figure 1.

The Before group included exhaled breath samples collected from patients with CRC at the time of primary diagnosis prior to surgical treatment. The After group consisted of paired exhaled breath samples obtained from the same patients on postoperative day 10 following radical surgical resection. The Control group comprised an independent cohort of healthy volunteers without a history of malignant disease or evidence of inflammatory or neoplastic bowel pathology at the time of enrollment. Healthy controls were frequency-matched to patients with CRC by age, sex, major comorbidities, and regular medication use. A single exhaled breath sample was collected from each healthy volunteer using the same standardized sampling protocol as that applied to patients with CRC.

The clinical and demographic characteristics of the final analytical cohort are presented in Table 1. The eligibility criteria, including inclusion, non-inclusion, and exclusion criteria for the CRC and control groups, are summarized in Table 2.

The study was approved by the Local Ethics Committee of I.M. Sechenov First Moscow State Medical University (Sechenov University) (Protocol No. 02-23, 26 January 2023). The study was conducted in accordance with the ethical principles of the Declaration of Helsinki. Written informed consent was obtained from all participants prior to study enrollment.

### 2.2. Breath Sample Collection

For patients in the Before group, breath samples were collected within 48 h prior to the scheduled radical surgical resection. All 38 patients included in the final paired analytical cohort had an uncomplicated postoperative course that permitted breath sampling on postoperative day 10.

Postoperative day 10 was selected as a standardized early postoperative sampling time point to characterize early postoperative changes in the exhaled volatilome within a consistent clinical timeframe; it was not intended to represent resolution of the physiological effects of surgery.

To standardize breath sampling conditions and minimize the influence of exogenous factors on VOC composition, all participants followed a predefined preparation protocol. Participants refrained from food intake, sweetened beverages, coffee, tea, and smoking for 8–12 h before sample collection. Consumption of plain drinking water was permitted throughout the fasting period. These measures were implemented to reduce preanalytical variability in breath VOC profiles and improve the reproducibility of the analytical results. However, postoperative factors including inflammatory responses, perioperative medication and antibiotic exposure, nutritional changes, gastrointestinal recovery, and microbiome alterations were not independently quantified or controlled and were therefore considered potential sources of residual confounding.

### 2.3. Breath Sample Processing and Predictor Space

Volatile organic compounds in exhaled breath were analyzed in real time using PTR-TOF-MS with an Ultra-Fast PTR-TOF 1000 mass spectrometer equipped with a Buffered End-Tidal Breath Sampling Inlet (IONICON Analytik GmbH, Innsbruck, Austria). Mass spectra were acquired in full-scan mode over a mass-to-charge ratio (*m*/*z*) range of 10–685 using H_3_O^+^ as the primary reagent ion. The scan time was 1000 ms, while the temperatures of both the drift tube (T-Drift) and sampling inlet (T-Inlet) were maintained at 80 °C throughout all measurements.

The acquired mass spectra were processed to identify individual ion peaks characterized by their mass-to-charge ratio (*m*/*z*) and normalized peak area. Raw mass spectrometry data were subjected to preprocessing to remove technical variables, including instrument metadata, calibration parameters, and quality control indices that were not related to the biological composition of the samples. The resulting analytical dataset consisted of 61 informative mass-spectral peaks. After removal of technical variables, all 61 remaining peaks constituted the initial candidate predictor set; no additional outcome-based or univariate pre-selection was performed before stability selection.

Peak areas were normalized during the preprocessing stage according to the standardized analytical workflow. Therefore, no additional normalization or distributional transformations were applied before downstream statistical analyses. Because exhaled breath was analyzed directly in real time using PTR-TOF-MS, discrete technical replicate injections were not performed. Analytical performance was monitored using routine instrumental calibration and quality-control procedures throughout the study.

### 2.4. Predictor Selection Strategy: Stability Selection

To address the challenges associated with a high-dimensional predictor space comprising 61 candidate VOC features relative to the limited cohort size (82 individuals: 38 patients contributing paired preoperative and postoperative samples and 44 independent healthy controls; 120 breath samples in total), a stability selection framework was employed to identify a robust subset of VOC features while reducing the risk of overfitting and unstable feature selection.

The stability selection procedure consisted of 1000 iterations of patient-grouped random subsampling using the GroupShuffleSplit algorithm. To prevent information leakage arising from subject-specific volatilome characteristics, the preoperative and postoperative breath samples from each patient were always assigned to the same partition (training or testing) within each iteration, ensuring that paired samples from the same patient never crossed the train–test boundary. Each healthy control was treated as an independent single-sample group. During each iteration, the dataset was randomly divided into training (50%) and testing (50%) subsets.

Within each iteration, all preprocessing and model-fitting steps were performed exclusively on the training subset. Candidate VOC features were standardized using the RobustScaler algorithm, which centers variables by their median and scales them according to the interquartile range, thereby reducing the influence of outliers commonly encountered in mass spectrometry data. To prevent data leakage, scaling parameters estimated from the training subset were subsequently applied without refitting to the corresponding testing subset.

Feature selection was performed using multinomial logistic regression with Elastic Net regularization (penalty = “elasticnet”, l1_ratio = 0.5, C = 0.1, solver = “saga”). To maintain model simplicity and avoid the instability that could arise from tuning multiple hyperparameters in the presence of a limited sample size, these regularization parameters were fixed a priori rather than tuned via an inner cross-validation loop. To account for class imbalance, inverse-frequency class weighting (class_weight = “balanced”) was incorporated during model training. During each iteration, candidate VOC features assigned non-zero regression coefficients for at least one of the three outcome classes were considered selected.

Following completion of all iterations, a stability score was calculated for each candidate VOC feature as the proportion of iterations in which the corresponding feature was retained by the model. The stability threshold was defined empirically as the 75th percentile of the observed stability score distribution derived from the current dataset. This data-driven approach retained features demonstrating consistently high selection frequencies within the present dataset. This empirical criterion was used as a dataset-specific feature-ranking threshold and should not be interpreted as a formal error-controlled stability selection threshold. The resulting threshold corresponded to a stability score of 0.621, yielding a final set of 16 VOC features.

### 2.5. Model Development and Performance Evaluation

The discriminative performance of the final predictor panel was evaluated using a one-versus-one (OvO) classification strategy for all pairwise comparisons among the three study groups: Before vs. After, Before vs. Control, and After vs. Control.

For each comparison, model development was embedded within the stability selection framework described above. During each of the 1000 patient-grouped train–test iterations, model training was performed using the training subset, whereas performance evaluation was conducted exclusively on the corresponding testing subset. To favor sensitivity within this exploratory classification framework, an adaptive probability-threshold optimization procedure was incorporated into the classification pipeline. Rather than applying the conventional probability threshold of 0.5, the optimal classification threshold was determined independently within each training subset as the lowest predicted probability achieving a predefined target sensitivity of at least 90%. The optimized threshold was subsequently applied without modification to the corresponding testing subset.

Model performance was assessed using accuracy, area under the receiver operating characteristic curve (ROC AUC), sensitivity, specificity, positive predictive value (PPV), and negative predictive value (NPV). Because the 1000 resampling iterations represent overlapping subsets of the same cohort rather than independent validation cohorts, performance estimates obtained across all iterations were summarized as the median together with the corresponding empirical 95% percentile range (95% PR; 2.5th–97.5th percentiles), rather than conventional confidence intervals. Model calibration and clinical utility were not assessed in this exploratory pilot analysis.

### 2.6. Comparative Statistical Analysis

To characterize the selected VOC features at the level of univariate distributions, pairwise comparisons of their signal intensities were performed between the study groups. Given the paired study design, differences between the preoperative and postoperative states (Before vs. After) were assessed using the non-parametric Wilcoxon signed-rank test. Comparisons involving the independent cohort of healthy volunteers (Before vs. Control and After vs. Control) were performed using the Mann–Whitney U test. To control the false discovery rate (FDR) associated with multiple comparisons, the Benjamini–Hochberg correction was applied separately within each pairwise contrast. All statistical tests were two-sided, and statistical significance was defined as an FDR-adjusted *p* value < 0.05.

Group distributions were summarized using medians and interquartile ranges (25th–75th percentiles). To provide a descriptive measure of the direction and magnitude of between-group differences, differences between group medians were additionally calculated and used for visualization of VOC signal patterns relative to healthy controls.

It should be noted that these univariate comparisons were performed exclusively on features previously selected through the data-driven stability selection procedure and should therefore be considered exploratory and descriptive rather than confirmatory. The reported *p* values, FDR-adjusted *p* values, and differences between group medians characterize the behavior of the selected VOC panel within the present cohort but may be subject to selection-induced bias. Accordingly, these analyses do not constitute confirmatory hypothesis testing across the entire initial predictor space, and independent external validation is required to confirm the observed univariate associations.

### 2.7. Reporting Standards and Reproducibility

The study design, definition of candidate predictors and target outcomes, validation strategy, and reporting of model performance were developed in accordance with the TRIPOD + AI (checklist version 11 January 2024) and STROBE reporting guidelines. All computational analyses were performed in Python 3.11 using the pandas, NumPy, scikit-learn, SciPy, statsmodels, NetworkX, and Matplotlib libraries; Matplotlib version 3.8.4 was used. The analytical code used for data preprocessing, statistical analyses, and model development is not publicly available.

## 3. Results

### 3.1. Study Population

A total of 50 patients with histologically confirmed stage II–III colorectal cancer were prospectively enrolled. Twelve patients were excluded because a valid postoperative day-10 breath sample was unavailable, resulting in a final paired CRC cohort of 38 patients with complete preoperative (Before) and postoperative day-10 (After) samples. An independent cohort of 44 healthy volunteers constituted the Control group. Thus, the final analytical dataset comprised 120 breath samples: 38 Before, 38 After, and 44 Control samples. Participant flow is summarized in Figure 1.

The mean age of patients with CRC was 62.3 ± 9.9 years, 22 (57.9%) were female, 11 (28.9%) had stage II disease, and 27 (71.1%) had stage III disease. The CRC and Control groups were comparable with respect to age, sex, body mass index, smoking status, major comorbidities, and regular medication use (all *p* > 0.05). Detailed demographic, clinical, tumor, and surgical characteristics are presented in Table 1.

### 3.2. Stability Selection Identified a 16-Feature VOC Panel

To derive a reproducibly selected panel of VOC features while accounting for repeated measurements within patients, a stability selection procedure based on 1000 iterations of patient-grouped random subsampling (GroupShuffleSplit) combined with multinomial logistic regression with Elastic Net regularization was performed. Preoperative and postoperative samples from the same patient were always assigned to the same train–test partition within each iteration, thereby preventing subject-level information leakage. Elastic Net regularization was selected as the base learner because it is well suited for high-dimensional datasets with correlated predictors and provides embedded feature selection through coefficient shrinkage.

Given the high dimensionality of the predictor space (61 candidate VOC features) relative to the available sample size (120 breath samples), a stability score was calculated for each candidate VOC feature as the proportion of resampling iterations in which the feature received a non-zero regression coefficient for at least one outcome class. The stability threshold was defined empirically as the 75th percentile of the observed stability score distribution derived from the current dataset. The resulting threshold corresponded to a stability score of 0.621, yielding a final set of 16 VOC features.

Application of the stability selection framework identified 16 VOC features (Table 3). The highest stability scores were observed for the ions with *m*/*z* 63.0157 (stability score = 0.999), 83.0849 (0.933), and 55.0395 (0.925). The remaining 13 selected VOC features demonstrated stability scores ranging from 0.621 to 0.914. The complete set of selected features and their corresponding stability scores is presented in Table 3.

### 3.3. Discriminative Performance of the Final VOC Panel

The discriminative performance of the final 16-VOC predictor panel was evaluated using a one-versus-one (OvO) strategy for all pairwise comparisons among the study groups: Before vs. After, Before vs. Control, and After vs. Control. An adaptive probability-threshold optimization procedure was incorporated into the evaluation framework. Within the training subset of each iteration, the lowest probability threshold achieving the predefined target sensitivity of ≥0.90 was identified and subsequently applied without modification to the corresponding test subset, thereby preventing the use of test-set information for threshold optimization. The discriminative performance of the final 16-VOC predictor panel is summarized in Table 4.

The highest median ROC AUC was observed for the After vs. Control comparison (AUC = 0.811), followed by Before vs. Control (AUC = 0.762) and Before vs. After (AUC = 0.716). All performance metrics were summarized across 1000 patient-grouped resampling iterations and are reported as median values with corresponding empirical 95% percentile ranges (95% PR; 2.5th–97.5th percentiles). Because these iterations represent overlapping resamples of the same cohort rather than independent validation cohorts, the reported percentile ranges should not be interpreted as conventional confidence intervals.

### 3.4. Interpretation of Model Discriminative Performance

The discriminative performance of the final 16-VOC predictor panel varied across the three pairwise classification tasks, with the lowest performance observed for discrimination between preoperative and postoperative samples.

#### 3.4.1. Preoperative vs. Control Discrimination (Before vs. Control)

Classification of treatment-naïve CRC patients (Before) and healthy controls demonstrated a median ROC AUC of 0.762 (95% PR, 0.629–0.869), with a median sensitivity of 0.714 and specificity of 0.706. These findings indicate moderate discrimination between patients with colorectal cancer and healthy individuals within the present cohort; however, this exploratory performance requires confirmation in an independent external validation cohort.

#### 3.4.2. Early Postoperative vs. Control Discrimination (After vs. Control)

Among the three pairwise comparisons, the After vs. Control comparison showed the highest discriminative performance, with a median ROC AUC of 0.811 (95% PR, 0.691–0.912), specificity of 0.824, and PPV of 0.822. These findings indicate that the early postoperative VOC profile remained distinguishable from that of healthy controls within the present exploratory dataset.

#### 3.4.3. Preoperative vs. Postoperative Discrimination (Before vs. After)

Discrimination between preoperative and postoperative samples was moderate, with a median ROC AUC of 0.716 (95% PR, 0.567–0.823). The median sensitivity was 0.737, whereas specificity was 0.632, with both metrics showing substantial variability across resampling iterations. The 95% PR for ROC AUC was relatively wide, indicating uncertainty in model performance for this comparison. Accordingly, these findings should be regarded as preliminary and suggest weak-to-moderate discriminatory ability of the current VOC panel for distinguishing the early postoperative state from the preoperative state. Further evaluation in larger independent cohorts and at additional postoperative time points is required before any clinical monitoring utility can be inferred.

### 3.5. Comparative Analysis of VOC Signal Intensities Between Study Groups

#### 3.5.1. Comparative Analysis of VOC Signal Intensities Across Clinical Groups

To further characterize the distributional differences of the selected VOC features, comparative analyses of VOC signal intensities were performed across the three study groups (Before, After, and Control). Pairwise group comparisons were conducted using non-parametric statistical tests, followed by Benjamini–Hochberg correction for multiple testing. For each VOC feature, median signal intensities, *p* values, FDR-adjusted *p* values, statistical significance, and differences between group medians were calculated.

Feature selection and univariate statistical testing addressed distinct analytical questions. The 16 VOC features were selected according to their contribution to the multivariable Elastic Net-based stability selection procedure and were not required to demonstrate statistically significant individual differences between study groups. Univariate statistical significance was therefore evaluated separately using pairwise tests with Benjamini–Hochberg FDR correction. The remaining selected feature, *m*/*z* 60.0553, differed significantly between the Before and Control groups but showed neither a significant paired Before vs. After difference nor a significant difference between the After and Control groups after FDR correction.

These univariate analyses should be considered exploratory and descriptive because they were performed on features previously identified through the data-driven stability selection procedure. The complete results of the pairwise comparisons are provided in Appendix A (Table A1).

Comparative analysis of the 16 VOC features selected by the stability selection procedure revealed heterogeneous patterns of signal intensities across the three study groups. Significant differences between the paired preoperative and early postoperative measurements were observed for five features (*m*/*z* 63.0157, 77.0576, 95.0534, 101.05, and 103.074; FDR-adjusted *p* < 0.05). In contrast, the remaining 11 selected features showed no significant differences between the Before and After measurements after FDR correction.

**Figure 2:**
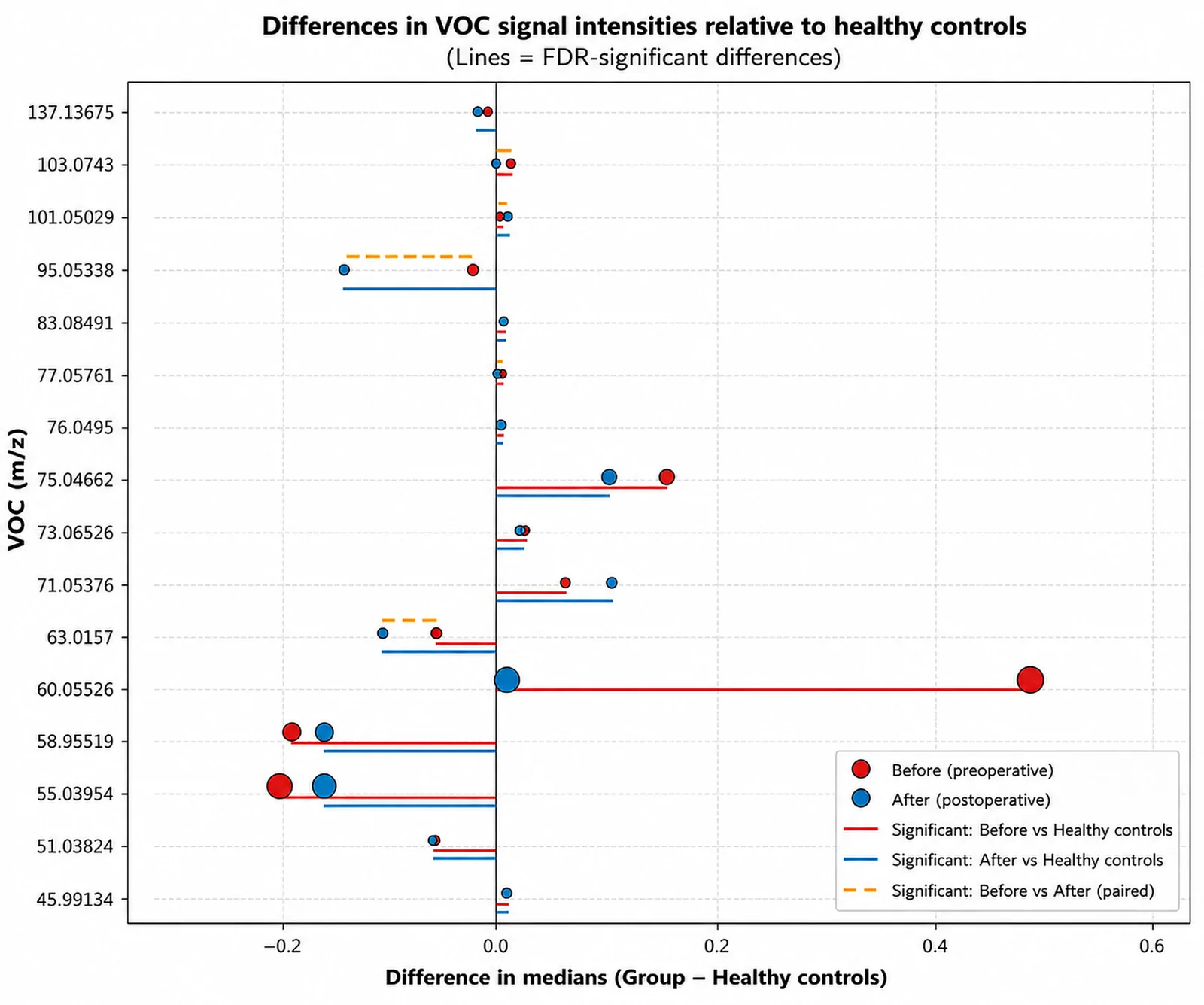
Differences in volatile organic compound signal intensities relative to healthy controls. The plot displays the median differences in normalized signal intensities between each clinical group and the healthy control group for the 16 selected VOC features (mass-to-charge ratio, *m*/*z*). Red points represent preoperative samples (Before group, n = 38), and blue points represent postoperative samples (After group, n = 38), with 44 healthy controls (Control group) serving as the reference. Coloured horizontal lines indicate statistically significant pairwise differences after Benjamini–Hochberg false discovery rate correction (FDR < 0.05): red lines denote significant differences between Before and Control groups, blue lines denote significant differences between After and Control groups, and orange dashed lines denote significant changes between Before and After groups (paired comparison). All signal intensities were normalized during the preprocessing stage of the analytical workflow as described in Section 2. Abbreviation: VOC, volatile organic compound.

Comparison with healthy controls demonstrated a broader pattern of between-group differences. Fourteen of the 16 selected VOC features differed significantly between the Before and Control groups, whereas two features (*m*/*z* 95.0534 and 137.137) showed no significant difference. At the early postoperative time point, 12 of the 16 selected features remained significantly different from Control, whereas four features (*m*/*z* 60.0553, 77.0576, 103.074, and 137.137) showed no significant difference after FDR correction. These findings indicate heterogeneous changes in individual VOC signal intensities across the preoperative, early postoperative, and healthy-control states. These patterns are visualized in Figure 2. Complete pairwise statistical results are provided in Appendix A (Table A1).

The visualization further demonstrates substantial heterogeneity in postoperative VOC changes. Several VOC features showed postoperative shifts in the direction of the signal intensities observed in healthy controls, whereas others remained largely unchanged or demonstrated only modest differences between the preoperative and early postoperative time points. These patterns should not be interpreted as evidence of metabolic normalization but rather as descriptive changes in individual VOC features during the early postoperative period. They formed the basis for the subsequent stratified presentation of pairwise statistical comparisons.

#### 3.5.2. Dynamic VOC Features Showing Significant Postoperative Changes

This subgroup comprises VOC features whose normalized signal intensities differed significantly between the preoperative and early postoperative time points. Five VOC features (*m*/*z* 63.0157, 77.0576, 95.0534, 101.05, and 103.074) demonstrated significant changes between the paired Before and After measurements after FDR correction (Table 5).

Among these features, *m*/*z* 77.0576 and 103.074 showed postoperative signal intensities that no longer differed significantly from those observed in healthy controls, whereas both features differed significantly from controls at the preoperative time point. In contrast, *m*/*z* 63.0157, 95.0534, and 101.05 remained significantly different from the healthy control group at the early postoperative time point despite exhibiting significant changes between the preoperative and postoperative measurements.

These findings demonstrate heterogeneous patterns of change among individual VOC features during the early postoperative period, with some features no longer differing significantly from healthy controls after surgery, while others remained significantly different.

#### 3.5.3. VOC Features Without Significant Preoperative-to-Postoperative Changes but with Persistent Differences from Controls

Nine selected VOC features did not demonstrate significant differences between the paired preoperative and early postoperative measurements after FDR correction but differed significantly from healthy controls at both time points. These included *m*/*z* 45.9913, 51.0382, 55.0395, 58.9552, 71.0538, 73.0653, 75.0466, 76.0495, and 83.0849.

For *m*/*z* 45.9913, 71.0538, 73.0653, 75.0466, 76.0495, and 83.0849, median signal intensities were higher in both the Before and After groups than in healthy controls. In contrast, *m*/*z* 51.0382, 55.0395, and 58.9552 showed lower median signal intensities in both patient time points relative to Control. Despite these persistent differences from healthy controls, none of these features demonstrated a statistically significant change between the preoperative and early postoperative measurements. Detailed pairwise comparisons are presented in Table 6.

#### 3.5.4. Stable VOC Features Without Significant Univariate Differences

Among the 16 VOC features retained by the stability selection procedure, *m*/*z* 137.137 did not demonstrate a statistically significant difference in normalized signal intensity in any of the three pairwise comparisons after FDR correction (Appendix A, Table A1). The FDR-adjusted *p* values were 0.504 for Before vs. After, 0.639 for Before vs. Control, and 0.050 for After vs. Control.

Despite the absence of statistically significant univariate differences, *m*/*z* 137.137 was retained in the final multivariable predictor panel by the Elastic Net-based stability selection procedure. This finding illustrates that multivariable feature selection and univariate statistical testing address distinct analytical questions and that inclusion of a feature in the multivariable panel does not necessarily require statistically significant individual differences between study groups.

## 4. Discussion

### 4.1. Principal Findings

One of the principal observations of the present study was the identification of five VOC features (*m*/*z* 63.0157, 77.0576, 95.0534, 101.05, and 103.074) that exhibited significant differences between the preoperative and early postoperative time points after FDR correction. Among these, *m*/*z* 77.0576 and 103.074 no longer differed significantly from healthy controls at the early postoperative time point, whereas *m*/*z* 63.0157, 95.0534, and 101.05 remained significantly different from controls despite significant preoperative-to-postoperative changes. These heterogeneous patterns indicate that early postoperative VOC changes do not follow a uniform trajectory and may reflect multiple biological and perioperative processes.

Importantly, the VOC changes observed on postoperative day 10 should not be interpreted as specific metabolic consequences of tumor removal. The early postoperative period encompasses multiple concurrent physiological and treatment-related processes, including surgical trauma, systemic inflammatory responses, wound healing, perioperative medication exposure, bowel preparation, altered nutritional intake, and acute perturbations of the gut microbiome. Each of these factors may influence the composition of exhaled VOCs. Therefore, the present findings are best interpreted as reflecting early postoperative remodeling of the exhaled volatilome following curative colorectal cancer surgery rather than tumor-specific metabolic normalization. The relative contribution of tumor removal to these changes cannot be determined from the present study design.

For *m*/*z* 77.0576 and 103.074, the absence of a statistically significant difference between the early postoperative and healthy control groups, despite significant differences between the preoperative and control groups, indicates a change in their statistical relationship with the control cohort following surgery. However, this pattern should not be interpreted as evidence of metabolic normalization or attributed specifically to tumor removal. Malignant tumors are known to influence systemic metabolism through multiple pathways, including altered glycolysis, oxidative stress, lipid metabolism, amino acid utilization, and interactions with the intestinal microbiome. At the same time, major abdominal surgery and early postoperative recovery are themselves associated with substantial physiological and metabolic perturbations that may influence the exhaled VOC profile.

The persistence of significant differences from healthy controls for *m*/*z* 63.0157, 95.0534, and 101.05 despite significant preoperative-to-postoperative changes further illustrates the heterogeneity of early postoperative VOC patterns. Early postoperative inflammation, wound healing, oxidative stress, transient alterations in gut microbial composition, and metabolic adaptation after major abdominal surgery may all contribute to sustained deviations in individual VOC features. Accordingly, the observed postoperative VOC trajectories are likely to reflect the combined influence of multiple concurrent processes rather than a simple transition from a cancer-associated volatilome to a healthy volatilome.

At the multivariable level, the After vs. Control comparison showed the highest discriminative performance (median ROC AUC = 0.811), followed by Before vs. Control (0.762), whereas Before vs. After showed the lowest discrimination (0.716). The Before vs. After comparison also demonstrated substantial variability across repeated resampling, indicating uncertainty in distinguishing the preoperative and early postoperative states. Importantly, the higher discrimination observed for After vs. Control should not be interpreted as evidence of postoperative metabolic normalization or as indicating a greater biological deviation from the healthy state. ROC AUC reflects multivariable group separability rather than the direction or biological meaning of changes in individual VOC features. Surgery-related physiological and perioperative changes may additionally contribute to the distinguishability of the early postoperative profile from that of healthy controls.

These findings highlight the potential value of paired breath analysis for characterizing temporal changes in the exhaled volatilome. However, with only two sampling time points and no non-oncological surgical control group, the present study cannot distinguish VOC changes potentially associated with tumor removal from those related to surgery and early postoperative recovery. The paired design nevertheless provides information on within-patient changes that cannot be obtained from cross-sectional comparisons alone and may therefore inform future longitudinal studies incorporating serial sampling beyond the early postoperative period and appropriate surgical control groups.

The observation that exhaled VOC profiles change following curative colorectal cancer surgery is consistent with previous studies and should not be regarded as unique to the present investigation. Altomare et al. reported postoperative alterations in exhaled VOC patterns using thermal-desorption GC-MS in disease-free patients during oncological follow-up [34], while Hanevelt et al. subsequently demonstrated short-term pre- to postoperative changes using an electronic-nose approach in a prospective paired cohort [35]. The present study extends these observations by applying real-time PTR-TOF-MS to paired preoperative and postoperative day-10 breath samples and by examining an exploratory multivariable signature composed of individual mass-to-charge features.

### 4.2. Biological Context of Early Postoperative VOC Changes

The early postoperative VOC changes identified in the present study demonstrate that the exhaled volatilome is dynamic across the perioperative period. Five VOC features showed significant differences between the preoperative and early postoperative measurements after FDR correction, although the biological processes underlying these changes cannot be determined from the present data. Previous studies have shown that exhaled VOC profiles may vary in association with malignant disease and therapeutic interventions [23,24,25,29,31,32,33,34,35]; however, in the present setting, the observed postoperative changes should be interpreted within the broader physiological context of major abdominal surgery and early recovery.

The biological origin of the detected VOC features is likely multifactorial. Colorectal cancer is associated with alterations in glucose, lipid, and amino acid metabolism, oxidative stress, and interactions with the intestinal microbiome, all of which may contribute to the composition of exhaled breath [9,10,11,12,13,16,21,22,41]. At the same time, surgical treatment and early postoperative recovery may independently modify systemic metabolism and gut microbial composition, potentially affecting VOC production [35,42]. The heterogeneous patterns observed among individual VOC features are therefore compatible with contributions from multiple biological and perioperative processes with potentially different temporal dynamics.

Importantly, PTR-TOF-MS provides high-resolution mass-to-charge features but does not establish definitive molecular identities. Individual *m*/*z* signals may represent different molecular species, fragments, cluster ions, or other contributions, and their biological origin cannot be inferred solely from accurate mass measurements. Accordingly, the VOC features identified in the present study should not be assigned to specific metabolic pathways on the basis of PTR-TOF-MS data alone. Orthogonal analytical confirmation, for example using GC-MS with authentic reference standards, will be required to establish their chemical identities and enable more specific mechanistic interpretation.

Taken together, these findings characterize a heterogeneous pattern of early postoperative volatilome remodeling rather than a tumor-specific metabolic response. The observed within-patient changes provide a basis for further investigation of temporal VOC dynamics following colorectal cancer surgery; however, determining whether particular VOC features are related to tumor removal, surgical recovery, or other perioperative processes will require serial sampling beyond the early postoperative period and appropriate surgical control groups.

#### Feature-Level Interpretation of Early Postoperative VOC Changes

Because PTR-TOF-MS detects mass-to-charge features rather than unequivocally identified compounds, the individual *m*/*z* signals observed in this study cannot be assigned to specific molecular species or metabolic pathways on the basis of accurate mass alone. Isomeric compounds, fragments, and cluster ions may contribute to the same detected feature, and definitive molecular characterization requires complementary chromatographic separation and confirmation using authentic reference standards [37,40]. Accordingly, the following discussion focuses on the observed preoperative-to-postoperative changes of the five VOC features rather than on compound-level or pathway-level assignments.

The feature at *m*/*z* 63.0157 showed a significant difference between the preoperative and early postoperative measurements and remained significantly different from healthy controls at the postoperative time point. Its molecular identity and biological origin cannot be determined from the present PTR-TOF-MS data. Therefore, the observed change should be interpreted at the feature level rather than as evidence of a specific metabolic process or pathway.

The feature at *m*/*z* 77.0576 showed a significant difference between the preoperative and early postoperative measurements. Although it differed significantly from healthy controls before surgery, no statistically significant difference was observed between the early postoperative and control groups. As its chemical identity was not established, the relevance of this feature in the present study lies in its observed pattern across the study groups rather than in a presumed molecular or biological assignment.

The feature at *m*/*z* 95.0534 changed significantly between the preoperative and early postoperative measurements but remained significantly different from healthy controls at the postoperative time point. Because the molecular identity of this feature has not been established, its postoperative behavior cannot be attributed to a specific metabolite, biological source, or metabolic pathway.

Similarly, *m*/*z* 101.05 showed a significant preoperative-to-postoperative change while remaining significantly different from healthy controls at the early postoperative time point. This finding indicates a change in the corresponding VOC feature during the early postoperative period but does not establish its chemical identity or biological origin. No specific mechanistic interpretation can therefore be assigned to this feature on the basis of the present data.

The feature at *m*/*z* 103.074 also showed a significant difference between the preoperative and early postoperative measurements. Whereas its preoperative signal intensity differed significantly from that of healthy controls, no statistically significant difference was observed between the early postoperative and control groups. Nevertheless, because chromatographic separation and confirmation using authentic reference standards were not performed, this signal cannot be attributed to a specific compound or metabolic pathway and should be interpreted solely as an analytically detected VOC feature.

Collectively, the heterogeneous patterns of these five VOC features demonstrate that several components of the exhaled volatilome changed between the preoperative and early postoperative time points. Two features (*m*/*z* 77.0576 and 103.074) no longer differed significantly from healthy controls at the early postoperative time point, whereas three (*m*/*z* 63.0157, 95.0534, and 101.05) remained significantly different from controls. However, because the detected features were not chemically identified, their behavior cannot establish specific metabolic mechanisms or biological sources. Furthermore, the observed postoperative changes cannot be attributed exclusively to tumor removal because the present study design does not distinguish tumor-related effects from other physiological and treatment-related processes occurring during early postoperative recovery. Chemical identification using complementary chromatographic techniques and authentic reference standards, together with longitudinal sampling beyond the early postoperative period, will be required to determine the molecular identity and biological significance of these features.

### 4.3. Biological Context of Persistent VOC Alterations

Several VOC features remained significantly different from healthy controls on postoperative day 10, indicating that differences in the exhaled volatilome persisted during the early postoperative period. Previous studies have similarly demonstrated short-term changes in exhaled VOC profiles following colorectal cancer surgery [35]. However, the persistence of differences in individual VOC features at this early time point should not be interpreted as evidence of incomplete tumor-related metabolic normalization, because the relative contributions of cancer-associated biology and perioperative physiological processes cannot be distinguished in the present study.

Persistent differences in VOC signal intensities may reflect biological processes that remain altered during early postoperative recovery. Colorectal cancer is associated with extensive metabolic and microbial alterations, while surgical treatment itself is accompanied by substantial changes in systemic metabolism, inflammatory activity, nutritional status, and gut microbial composition [42]. In addition, postoperative inflammation, oxidative stress, tissue repair, dietary changes, bowel preparation, and perioperative medication exposure may contribute to sustained differences in individual VOC features during this period.

The direction of these persistent differences should likewise be interpreted cautiously. Features that remained either higher or lower than those observed in healthy controls may reflect differences in multiple host-, microbial-, or perioperative processes, but their molecular origins cannot be established from the present PTR-TOF-MS data. Previous studies have demonstrated reproducible alterations in the gut microbiome associated with colorectal cancer across different populations [43]; however, whether such microbial changes contributed to the persistent VOC features observed in the present cohort cannot be determined without direct microbiome and chemical analyses.

Importantly, persistent postoperative differences in VOC features should not be interpreted as direct evidence of residual disease. At present, they should be considered characteristics of the early postoperative volatilome whose biological determinants remain uncertain. Serial sampling beyond the postoperative recovery period will be required to determine whether these differences are transient, diminish over time, or persist during longer follow-up. Only subsequent longitudinal studies incorporating clinical outcomes could establish whether any of these features have potential prognostic relevance for postoperative surveillance or recurrence assessment.

### 4.4. Multivariable VOC Signatures Beyond Univariate Analysis

Not all VOC features retained in the final predictor panel demonstrated statistically significant differences in univariate comparisons. This is not unexpected because multivariable feature selection and univariate statistical testing address distinct analytical questions. Breath represents an integrated metabolic phenotype in which multiple correlated changes may collectively contribute to discrimination between clinical states even when individual features do not demonstrate statistically significant between-group differences. Accordingly, breath-based classification in the present study is better conceptualized as multivariable pattern recognition than as the identification of individual disease-specific metabolites.

Elastic Net regression was selected because it combines regularization with embedded feature selection and can retain information from correlated predictors, a relevant property for high-dimensional VOC data. In contrast to more complex nonlinear algorithms, it also provides a comparatively transparent predictor structure and was considered appropriate for the dimensionality, correlation structure, and sample size of the present dataset [44]. This choice should not be interpreted as evidence that Elastic Net is universally superior to alternative machine-learning approaches, such as Random Forest or gradient-boosting methods. Repeated subsampling-based stability selection was additionally used to identify features that were consistently retained across different data partitions. By prioritizing VOC features repeatedly selected across resampling iterations, this procedure was intended to reduce dependence of feature selection on any single data split. Nevertheless, stability selection does not eliminate the risks of model instability or overfitting, particularly in a dataset of the present size, and the resulting predictor panel requires validation in an independent external cohort [45].

The retention of VOC features without statistically significant univariate differences further illustrates that the predictive information captured by the model may arise from the joint contribution of multiple features rather than from isolated statistically significant signals. The resulting 16-feature panel should therefore be regarded as an exploratory multivariable signature rather than as a set of individually validated biomarkers. Transparent reporting of the complete modeling and evaluation process is essential for interpreting these findings and facilitating subsequent external validation, consistent with the principles of TRIPOD + AI [46].

### 4.5. Clinical Implications

Exhaled VOC profiling offers a non-invasive and repeatable approach for characterizing changes in the breath volatilome over time. In the present study, measurable within-patient changes were observed between the preoperative and early postoperative time points; however, these changes cannot be attributed specifically to tumor removal and should not be interpreted as markers of treatment response, residual disease, or recurrence. Accordingly, the identified multivariable VOC signature should be regarded as exploratory and as characterizing early postoperative volatilome changes rather than as a validated tool for postoperative monitoring or oncological surveillance. The present findings therefore support further investigation of serial breath analysis for characterizing postoperative VOC dynamics [47].

The potential translational relevance of PTR-TOF-MS may lie in its capacity for repeated longitudinal assessment rather than in its immediate application as a diagnostic or surveillance test. Before any clinical implementation can be considered, the identified multivariable VOC signature requires independent external validation, standardized sampling and analytical procedures, assessment of model calibration and clinical utility, and comparison with established surveillance methods. Future prospective studies should incorporate serial sampling beyond the early postoperative period, longer oncological follow-up, and appropriate non-oncological surgical control groups to determine whether specific patterns of VOC change are associated with tumor-related processes rather than with surgery and postoperative recovery. At present, breath analysis should be regarded as an investigational approach rather than an established component of postoperative colorectal cancer surveillance [23,25,29,32].

### 4.6. Strengths and Limitations

This study has several strengths. First, breath samples were collected prospectively using a paired design, allowing direct assessment of within-patient changes in VOC profiles between the preoperative and early postoperative time points. Second, PTR-TOF-MS enabled real-time, high-resolution detection of exhaled VOC features with minimal sample preparation. Third, the study integrated univariate analyses with regularized multivariable modeling and repeated subsampling to explore VOC patterns beyond individual feature-level differences.

Several limitations should also be acknowledged. First, this was a single-center pilot study with a limited sample size. The relatively small number of patients, particularly in relation to the number of candidate predictors included in the multivariable analysis, increases the risk of model instability and overfitting and limits the precision and generalizability of the estimated discriminatory performance. The limited sample size and unequal distribution of patients across tumor stage, anatomical location, and surgical-procedure subgroups precluded reliable stratified analyses of these clinical factors. In addition, 12 of the 50 initially enrolled patients were excluded from the final paired analysis because a valid postoperative day-10 breath sample was unavailable. Although the reasons for exclusion were documented, including refusal of repeat sampling, postoperative complications, and technically unsuitable samples, the exclusion of patients without complete paired data may have introduced attrition-related selection bias, particularly because postoperative complications precluded day-10 sampling in three patients. The identified multivariable VOC signature should therefore be considered exploratory and requires validation in larger, independent external cohorts. In addition, the restrictive eligibility criteria and the selected nature of the final paired cohort may limit the generalizability of the findings to the broader CRC population, particularly to patients with greater comorbidity burden or complicated postoperative recovery.

Second, postoperative breath sampling was performed at a single early time point, on postoperative day 10. At this stage, the effects of tumor removal cannot be separated from those of major abdominal surgery and early postoperative recovery. Surgical trauma, systemic inflammation, wound healing, anesthesia, perioperative medication exposure including antibiotic therapy, bowel preparation, altered nutritional intake, and acute perturbations of the gut microbiome may all contribute to the observed VOC changes.

The absence of a non-oncological abdominal surgical control group further limits causal attribution of postoperative VOC changes to tumor removal. Serial sampling beyond the early recovery period, together with appropriate surgical control groups, will be required to distinguish potentially tumor-related changes from nonspecific perioperative effects.

Third, breath VOC profiles may be influenced by physiological, behavioral, and clinical factors, including comorbidities, diet, medication exposure, smoking status, and microbiome composition. Although standardized sampling procedures were applied to reduce preanalytical variability, the study was not designed to quantify the independent effects of these factors, and residual confounding cannot be excluded. In particular, the independent contribution of comorbidities to the observed VOC patterns cannot be completely excluded.

Fourth, PTR-TOF-MS provides high-resolution mass-to-charge measurements but does not establish definitive molecular identities or distinguish all potential isomers, fragments, and cluster ions contributing to individual signals. Consequently, the detected signals should be interpreted as VOC features rather than unequivocally identified compounds [40]. Orthogonal chemical confirmation, for example using chromatographic separation coupled with mass spectrometry and authentic reference standards, will be necessary before specific molecular identities or metabolic pathways can be assigned to these features. Detailed quantitative assessments of analytical variability, including technical coefficients of variation and batch-drift effects, were not available for the present analysis. In addition, background-air, blank, and humidity-related effects were not systematically quantified. These analytical quality-control procedures should be incorporated into future validation studies.

In addition, the observed VOC changes were not independently corroborated by direct biochemical or metabolomic measurements, which further limits mechanistic interpretation of the detected features.

Finally, the multivariable model was developed and internally evaluated within the same single-center cohort and has not undergone independent external validation. Accordingly, the reported discriminatory performance should be considered preliminary and hypothesis-generating rather than evidence of a clinically validated predictive model. Model calibration, clinical utility, and performance in independent populations remain to be established. In addition, formal optimism correction beyond the repeated internal resampling procedure was not performed. Long-term postoperative VOC trajectories and their potential association with recurrence or other clinical outcomes also remain unknown.

## 5. Conclusions

This study identified measurable changes in the exhaled VOC profile between the preoperative and early postoperative time points following curative colorectal cancer surgery. Five VOC features demonstrated significant differences between the paired preoperative and early postoperative measurements after FDR correction. Among these, some no longer differed significantly from healthy controls at the early postoperative time point, whereas others remained significantly different, demonstrating heterogeneous patterns of early postoperative volatilome remodeling. However, given the timing of postoperative sampling and the absence of a non-oncological surgical control group, these changes cannot be attributed specifically to tumor removal and may reflect the combined influence of multiple biological and perioperative processes.

These findings support further investigation of PTR-TOF-MS-based breath analysis as a non-invasive approach for characterizing longitudinal changes in the exhaled volatilome following colorectal cancer surgery. The observed heterogeneity across individual VOC features further supports investigation of multivariable breath signatures; however, the identified 16-feature signature should be considered exploratory and requires independent external validation. The present findings do not establish its ability to assess treatment response, detect residual disease, predict recurrence, or support clinical surveillance. Future studies incorporating larger multicenter cohorts, serial sampling beyond the early postoperative period, appropriate surgical control groups, and orthogonal chemical identification are needed to determine whether longitudinal VOC patterns can provide clinically relevant information during postoperative follow-up.

## Figures and Tables

**Table 1:** Participant characteristics.

Characteristic	CRC Patients (*n* = 38)	Healthy Controls (*n* = 44)	*p*-Value
Age, years, mean ± SD	62.3 ± 9.9	62.3 ± 5.6	0.993
**Sex**			
Male	16 (42.1)	14 (31.8)	0.366
Female	22 (57.9)	30 (68.2)	0.366
Body mass index, kg/m^2^, mean ± SD	26.0 ± 4.3	26.0 ± 2.1	0.991
Current smoking status	3 (7.9)	7 (15.9)	0.326
**Comorbidities**			
Hypertension	12 (31.6)	8 (18.2)	0.201
Coronary heart disease ^1^	5 (13.2)	10 (22.7)	0.391
Upper gastrointestinal disease ^2^	10 (26.3)	10 (22.7)	0.799
Iron-deficiency anemia	3 (7.9)	1 (2.3)	0.332
**Regular medication**			
Antihypertensive therapy	12 (31.6)	8 (18.2)	0.201
Statins	9 (23.7)	5 (11.4)	0.155
Proton pump inhibitors	7 (18.4)	7 (15.9)	0.777
Diuretics	5 (13.2)	2 (4.5)	0.241
Iron supplementation	1 (2.6)	1 (2.3)	1.000
Antiplatelet agents	3 (7.9)	5 (11.4)	0.719
**Surgical treatment**			
Right hemicolectomy	8 (21.1)	n/a	n/a
Left hemicolectomy	1 (2.6)	n/a	n/a
Sigmoid colectomy	16 (42.1)	n/a	n/a
Rectal resection ^3^	13 (34.2)	n/a	n/a
**Primary tumor location**			
Cecum	3 (7.9)	n/a	n/a
Ascending colon	1 (2.6)	n/a	n/a
Hepatic flexure	3 (7.9)	n/a	n/a
Transverse colon	1 (2.6)	n/a	n/a
Descending colon	1 (2.6)	n/a	n/a
Sigmoid colon	16 (42.1)	n/a	n/a
Rectosigmoid junction	5 (13.2)	n/a	n/a
Rectum	8 (21.1)	n/a	n/a
**Clinical stage**			
Stage II	11 (28.9)	n/a	n/a
Stage III	27 (71.1)	n/a	n/a
**Histological grade**			
Low-grade (G1–G2)	37 (97.4)	n/a	n/a
High-grade (G3)	1 (2.6)	n/a	n/a

Data are presented as mean ± standard deviation (SD) or n (%). *p*-values are provided for descriptive purposes only and should be interpreted in the context of the frequency-matched study design. Healthy controls were frequency-matched to patients with colorectal cancer by age, sex, major comorbidities, and regular medication use. ^1^ Includes documented coronary artery disease and/or aortic/coronary atherosclerosis. ^2^ Includes chronic gastritis, duodenitis, and gastroesophageal reflux disease. ^3^ Rectal resection includes anterior resection, low anterior resection, ultra-low anterior resection, abdominal–anal resection, abdominoperineal excision, sphincter-preserving rectal resections, and other rectal resections.

**Table 2:** Eligibility criteria for patient enrollment in the study.

Category	Eligibility Criteria
**Inclusion criteria**	Written informed consent obtained prior to study enrollment. Adult patients (≥18 years of age). Patients scheduled to undergo primary radical surgical treatment for colorectal cancer according to the Russian Ministry of Health clinical practice guidelines. Histologically confirmed colorectal cancer (clinical stage II–III). Completion of the standard laboratory and instrumental diagnostic work-up according to national clinical guidelines.
**Non-inclusion criteria**	Severe concomitant diseases that could substantially affect systemic metabolism or breath VOC composition, including heart failure (left ventricular ejection fraction <50% and/or NYHA class III–IV), chronic kidney disease (eGFR < 60 mL/min/1.73 m^2^), and hepatic impairment (Child–Pugh class A–C) ^1^. Cystic fibrosis. Chronic pulmonary diseases. Systemic inflammatory diseases, including connective tissue disorders, rheumatologic diseases, and systemic vasculitis. Terminal-stage malignancy or palliative treatment. Hematologic disorders, including lymphoproliferative diseases. Multiple primary malignant neoplasms. Previous chemotherapy and/or radiotherapy. Chronic alcohol abuse. Active alcohol or drug dependence that could interfere with study participation. Severe psychiatric disorders that could interfere with informed consent, protocol compliance, or study participation. Type 1 or type 2 diabetes mellitus.
**Exclusion criteria**	Unavailability of either the preoperative or postoperative breath sample required for the paired VOC analysis. Withdrawal of consent or refusal to continue participation in the study. Active inflammatory conditions, including acute infections or exacerbation of chronic inflammatory diseases, that could substantially affect systemic metabolism or breath VOC composition ^2^.

^1^ These criteria were selected to minimize the influence of impaired cardiac, renal, and hepatic function on systemic metabolism and the composition of exhaled volatile organic compounds, thereby reducing potential confounding effects unrelated to colorectal cancer. ^2^ Active inflammatory conditions, including acute infections or exacerbation of chronic inflammatory diseases, acute thromboembolic events, that could substantially affect systemic metabolism or breath VOC composition.

**Table 3:** Stability selection frequencies of the 61 candidate VOC features across 1000 patient-grouped resampling iterations.

Feature (*m*/*z*)	Stability Score
**63.0157**	**0.999**
**83.0849**	**0.933**
**55.0395**	**0.925**
**45.9913**	**0.914**
**51.0382**	**0.91**
**103.074**	**0.89**
**60.0553**	**0.824**
**71.0538**	**0.803**
**95.0534**	**0.799**
**101.05**	**0.765**
**58.9552**	**0.751**
**137.137**	**0.719**
**75.0466**	**0.685**
**76.0495**	**0.653**
**73.0653**	**0.651**
**77.0576**	**0.621**
121.093	0.596
59.0603	0.557
65.0587	0.536
118.07	0.519
91.0531	0.491
74.0477	0.48
69.0725	0.474
89.0605	0.47
107.089	0.461
373.088	0.451
93.0677	0.435
371.101	0.424
111.113	0.415
357.077	0.41
97.1	0.394
117.078	0.386
70.0752	0.366
79.0529	0.364
372.101	0.345
105.059	0.332
61.0328	0.322
90.0644	0.319
109.093	0.307
331.853	0.294
44.048	0.279
87.0734	0.267
329.84	0.232
62.0311	0.213
99.0639	0.202
43.0468	0.156
90.9507	0.153
115.096	0.126
57.0688	0.124
47.0403	0.087
355.073	0.074
67.0563	0.07
44.9911	0.059
68.0622	0.051
81.0722	0.034
48.0444	0.034
356.076	0.033
53.0371	0.002
203.949	0
330.845	0
204.952	0

Stability score represents the proportion of the 1000 patient-grouped resampling iterations in which a given VOC feature was retained by the Elastic Net model with a non-zero regression coefficient for at least one outcome class. The stability threshold was defined empirically as the 75th percentile of the observed stability score distribution (0.621). Features meeting or exceeding this threshold were included in the final 16-feature panel and are shown in bold. VOC, volatile organic compound; *m*/*z*, mass-to-charge ratio.

**Table 4:** Discriminative performance of the final 16-VOC panel across 1000 patient-grouped resampling iterations.

Performance Metric	Before vs. After	Before vs. Control	After vs. Control
**Accuracy**	0.674 [0.543–0.806]	0.707 [0.561–0.829]	0.756 [0.610–0.854]
**ROC AUC**	0.716 [0.567–0.823]	0.762 [0.629–0.869]	0.811 [0.691–0.912]
**Sensitivity**	0.737 [0.409–0.952]	0.714 [0.435–0.950]	0.714 [0.435–0.950]
**Specificity**	0.632 [0.211–0.917]	0.706 [0.363–0.947]	0.824 [0.400–1.000]
**Positive Predictive Value (PPV)**	0.667 [0.526–0.867]	0.735 [0.550–0.938]	0.822 [0.583–1.000]
**Negative Predictive Value (NPV)**	0.700 [0.536–0.923]	0.682 [0.500–0.900]	0.708 [0.524–0.909]

Data are presented as median [95% percentile range (95% PR)] across 1000 patient-grouped resampling iterations. The 95% PR corresponds to the 2.5th–97.5th percentiles of the empirical distribution of performance estimates across repeated train–test splits and should not be interpreted as a conventional confidence interval. Probability thresholds were optimized within each training subset to achieve a predefined target sensitivity of ≥0.90 and were subsequently applied without modification to the corresponding test subset. PPV and NPV depend on the class proportions in the present study sample and therefore should not be interpreted as population-level predictive values. ROC AUC, area under the receiver operating characteristic curve; PPV, positive predictive value; NPV, negative predictive value.

**Table 5:** Pairwise comparisons of VOC features showing significant differences between the preoperative and early postoperative time points.

VOC (*m*/*z*)	Before (n = 38)	After (n = 38)	Control (n = 44)	Before vs. After FDR-Adjusted *p*	Before vs. Control FDR-Adjusted *p*	After vs. Control FDR-Adjusted *p*
63.0157	0.150 [0.120, 0.227]	0.103 [0.089, 0.124]	0.200 [0.150, 0.436]	<0.001	0.024	<0.001
77.0576	0.031 [0.025, 0.043]	0.027 [0.020, 0.032]	0.026 [0.020, 0.031]	0.033	0.007	0.739
95.0534	0.160 [0.089, 0.352]	0.046 [0.025, 0.129]	0.180 [0.119, 0.257]	<0.001	0.777	<0.001
101.05	0.017 [0.014, 0.025]	0.023 [0.019, 0.028]	0.014 [0.012, 0.017]	0.033	0.007	<0.001
103.074	0.029 [0.018, 0.042]	0.016 [0.012, 0.023]	0.017 [0.013, 0.021]	0.017	<0.001	0.959

Data are presented as median [25th, 75th percentiles] of normalized VOC signal intensities. Only VOC features with statistically significant differences between the paired Before and After measurements after FDR correction are included. Before vs. After comparisons were performed using the Wilcoxon signed-rank test, whereas Before vs. Control and After vs. Control comparisons were performed using the Mann–Whitney U test. *p* values were adjusted for multiple comparisons within each pairwise contrast using the Benjamini–Hochberg false discovery rate (FDR) procedure. Statistical significance was defined as an FDR-adjusted *p* value < 0.05. VOC, volatile organic compound; *m*/*z*, mass-to-charge ratio; FDR, false discovery rate.

**Table 6:** VOC features without significant preoperative-to-postoperative changes but with persistent differences from healthy controls.

VOC (*m*/*z*)	Before (n = 38)	After (n = 38)	Control (n = 44)	Before vs. After FDR-Adjusted *p*	Before vs. Control FDR-Adjusted *p*	After vs. Control FDR-Adjusted *p*
45.9913	0.056 [0.047, 0.074]	0.057 [0.051, 0.066]	0.048 [0.041, 0.062]	0.989	0.036	0.005
51.0382	0.039 [0.031, 0.055]	0.037 [0.032, 0.053]	0.092 [0.062, 0.121]	0.989	<0.001	<0.001
55.0395	0.783 [0.635, 0.904]	0.824 [0.694, 0.920]	0.981 [0.786, 1.142]	0.574	0.005	0.016
58.9552	0.414 [0.305, 0.585]	0.444 [0.372, 0.523]	0.601 [0.436, 0.678]	0.989	0.024	0.012
71.0538	0.111 [0.064, 0.248]	0.150 [0.089, 0.267]	0.051 [0.032, 0.096]	0.989	<0.001	<0.001
73.0653	0.070 [0.046, 0.085]	0.066 [0.054, 0.091]	0.044 [0.037, 0.051]	0.989	<0.001	<0.001
75.0466	0.352 [0.257, 0.777]	0.298 [0.180, 0.831]	0.203 [0.118, 0.367]	0.989	0.003	0.012
76.0495	0.020 [0.015, 0.035]	0.019 [0.013, 0.037]	0.015 [0.012, 0.019]	0.989	0.005	0.048
83.0849	0.018 [0.012, 0.033]	0.019 [0.014, 0.030]	0.012 [0.010, 0.019]	0.989	0.036	0.012

Data are presented as median [25th, 75th percentiles] of normalized VOC signal intensities. Only VOC features without a statistically significant difference between the paired Before and After measurements after FDR correction, but with statistically significant differences from healthy controls at both time points, are included. Before vs. After comparisons were performed using the Wilcoxon signed-rank test, whereas Before vs. Control and After vs. Control comparisons were performed using the Mann–Whitney U test. *p* values were adjusted for multiple comparisons within each pairwise contrast using the Benjamini–Hochberg false discovery rate (FDR) procedure. Statistical significance was defined as an FDR-adjusted *p* value < 0.05. VOC, volatile organic compound; *m*/*z*, mass-to-charge ratio; FDR, false discovery rate.

## Data Availability

Data are available from the corresponding author upon reasonable request.

## Abbreviations

The following abbreviations are used in this manuscript:

CRC	Colorectal Cancer
CT	Computed Tomography
DNA	Deoxyribonucleic Acid
FDR	False Discovery Rate
FIT	Fecal Immunochemical Test
IQR	Interquartile Range
MRI	Magnetic Resonance Imaging
NPV	Negative Predictive Value
OvO	One-versus-One
PET/CT	Positron Emission Tomography/Computed Tomography
PPV	Positive Predictive Value
PTR-TOF-MS	Proton Transfer Reaction Time-of-Flight Mass Spectrometry
ROC	Receiver Operating Characteristic
STROBE	Strengthening the Reporting of Observational Studies in Epidemiology
TRIPOD + AI	Transparent Reporting of a Multivariable Prediction Model for Individual Prognosis or Diagnosis–Artificial Intelligence
VOCs	Volatile Organic Compounds

## Appendix A

**Table A1:** Pairwise comparisons of normalized signal intensities for the selected VOC features across the study groups.

VOC (*m*/*z*)	Before (n = 38)	After (n = 38)	Control (n = 44)	Before vs. After *p*	Before vs. After FDR	Before vs. After Significance	Before vs. Control *p*	Before vs. Control FDR	Before vs. Control Significance	After vs. Control *p*	After vs. Control FDR	After vs. Control Significance
63.0157	0.150 [0.120, 0.227]	0.103 [0.089, 0.124]	0.200 [0.150, 0.436]	<0.001	<0.001	Yes	0.018	0.024	Yes	<0.001	<0.001	Yes
83.0849	0.018 [0.012, 0.033]	0.019 [0.014, 0.030]	0.012 [0.010, 0.019]	0.796	0.989	No	0.031	0.036	Yes	0.007	0.012	Yes
55.0395	0.783 [0.635, 0.904]	0.824 [0.694, 0.920]	0.981 [0.786, 1.142]	0.287	0.574	No	0.002	0.005	Yes	0.011	0.016	Yes
45.9913	0.056 [0.047, 0.074]	0.057 [0.051, 0.066]	0.048 [0.041, 0.062]	0.657	0.989	No	0.030	0.036	Yes	0.002	0.005	Yes
51.0382	0.039 [0.031, 0.055]	0.037 [0.032, 0.053]	0.092 [0.062, 0.121]	0.785	0.989	No	<0.001	<0.001	Yes	<0.001	<0.001	Yes
103.074	0.029 [0.018, 0.042]	0.016 [0.012, 0.023]	0.017 [0.013, 0.021]	0.003	0.017	Yes	<0.001	<0.001	Yes	0.959	0.959	No
60.0553	1.268 [0.717, 2.171]	0.795 [0.476, 1.283]	0.787 [0.537, 1.115]	0.090	0.240	No	0.014	0.023	Yes	0.693	0.739	No
71.0538	0.111 [0.064, 0.248]	0.150 [0.089, 0.267]	0.051 [0.032, 0.096]	0.989	0.989	No	<0.001	<0.001	Yes	<0.001	<0.001	Yes
95.0534	0.160 [0.089, 0.352]	0.046 [0.025, 0.129]	0.180 [0.119, 0.257]	<0.001	<0.001	Yes	0.777	0.777	No	<0.001	<0.001	Yes
101.05	0.017 [0.014, 0.025]	0.023 [0.019, 0.028]	0.014 [0.012, 0.017]	0.010	0.033	Yes	0.004	0.007	Yes	<0.001	<0.001	Yes
58.9552	0.414 [0.305, 0.585]	0.444 [0.372, 0.523]	0.601 [0.436, 0.678]	0.830	0.989	No	0.017	0.024	Yes	0.007	0.012	Yes
137.137	0.027 [0.018, 0.039]	0.019 [0.014, 0.031]	0.034 [0.015, 0.053]	0.220	0.504	No	0.599	0.639	No	0.040	0.050	No
75.0466	0.352 [0.257, 0.777]	0.298 [0.180, 0.831]	0.203 [0.118, 0.367]	0.931	0.989	No	<0.001	0.003	Yes	0.006	0.012	Yes
76.0495	0.020 [0.015, 0.035]	0.019 [0.013, 0.037]	0.015 [0.012, 0.019]	0.954	0.989	No	0.002	0.005	Yes	0.036	0.048	Yes
73.0653	0.070 [0.046, 0.085]	0.066 [0.054, 0.091]	0.044 [0.037, 0.051]	0.657	0.989	No	<0.001	<0.001	Yes	<0.001	<0.001	Yes
77.0576	0.031 [0.025, 0.043]	0.027 [0.020, 0.032]	0.026 [0.020, 0.031]	0.010	0.033	Yes	0.004	0.007	Yes	0.665	0.739	No

Data are presented as median [25th, 75th percentiles] of normalized VOC signal intensities. Comparisons between paired Before and After measurements were performed using the Wilcoxon signed-rank test, whereas Before vs. Control and After vs. Control comparisons were performed using the Mann–Whitney U test. *p* values were adjusted for multiple comparisons within each pairwise contrast using the Benjamini–Hochberg false discovery rate (FDR) procedure. Statistical significance was defined as an FDR-adjusted *p* value < 0.05. VOC, volatile organic compound; *m*/*z*, mass-to-charge ratio; FDR, false discovery rate.
